# The Value of Technetium-99m Labeled Alpha-Melanocyte-Stimulating Hormone (^99m^Tc-α-MSH) in Diagnosis of Primary and Metastatic Lesions of Malignant Melanoma 

**DOI:** 10.22038/aojnmb.2018.30101.1204

**Published:** 2018

**Authors:** Saeed Farzanefar, Rahman Etemadi, Mohammad Shirkhoda, Habibollah Mahmoodzadeh, Mostafa Erfani, Babak Fallahi, Mehrshad Abbasi, Narjess Ayati, Arman Hassanzadeh-Rad, Mohammad Eftekhari, Davood Beiki

**Affiliations:** 1 Department of Nuclear Medicine, Vali-Asr Hospital, Tehran University of Medical Sciences, Tehran, Iran; 2 Research Center for Nuclear Medicine, Shariati Hospital, Tehran University of Medical Sciences, Tehran, Iran; 3 Department of General Surgery, Cancer Research Center, Cancer Institute, Tehran University of Medical Sciences, Tehran, Iran; 4 Radiation Application Research School, Nuclear Science and Technology Research Institute, Tehran, Iran; 5 Nuclear Medicine Research Center, Mashhad University of Medical Sciences, Mashhad, Iran

**Keywords:** Malignant Melanoma, Melanocortine-1 receptor, Melanocyte stimulating hormone, Radiolabeled peptide, ^99m^Tc-α-MSH

## Abstract

**Objective(s):**

Malignant melanoma is the most lethal type of skin cancers with unfavorable prognosis. Alpha-MSH peptide analogues have a high affinity for melanocortine-1 (MC1) receptors on melanocytes over expressing in malignant melanoma cells. Pre-clinical studies have shown promising results for radiolabeled MSH imaging in this malignancy. The purpose of this study is to assess the diagnostic value of ^99m^Tc-α-MSH imaging in malignant melanoma.

**Methods:**

Twenty-one patients (13 men) with pathologically confirmed malignant melanoma with or without metastatic distribution were included in this study. 740-1110 MBq ^99m^Tc-α-MSH was injected and whole body scans were performed 20, 120 and 240 minutes post injection and were assessed both qualitatively and semi-quantitatively using target (T) to background (BG) ratio.

**Results:**

The T/BG ratio for the primary tumor bed was 2.51±2.26, 2.56±2.48 and 1.92±1.79 minutes in the whole body scans 20, 120 and 240 minutes post injection, respectively. The sensitivity, specificity, negative and positive predictive values were 75%, 80%, 50% and 92% for primary lesion and 25%, 100%, 68% and 100% for distant metastasis, respectively.

**Conclusion:**

^99m^Tc-α-MSH is a newly introduced agent for diagnosis of tumoral lesions in malignant melanoma. Our study showed a high sensitivity with this modality in primary lesions as well as lymph node involvements. However the detection rate was not high in distant metastasis. The preliminary results are promising especially as a new complementary imaging method in management of malignant melanoma.

## Introduction

Malignant melanoma is the most lethal type of skin cancers originating from melanocytes which are located in the basal layer of the epidermis (1). The incidence of this poor prognosis pathology is increasing in recent years. As the metastatic melanoma has very poor outcome with less than one year average survival after metastatic dissemination (2), it is highly important to diagnose this malignancy in early curable stages. In addition, in case of thick melanoma a whole body survey to accurately detect every possible metastatic lesion is mandatory for staging, restaging and treatment planning (3).

Currently, ^18^F-FDG PET/CT is the modality of choice for cancer staging in variety of malignancies including melanoma (4). As the tumoral cells show higher level of metabolism, increased accumulation of ^18^F-FDG in tumoral cells can be detected this imaging method. On the other hand, ^18^F-FDG PET/CT is a non-specific imaging modality and at the same time, there are some kinds of malignant melanoma with no detectability with ^18^F-FDG PET (5), therefore, finding a radiolabeled specific agent to dedicatedly bind to melanocytes is highly desirable. Αlpha-melanocyte-stimulating hormone (α-MSH) peptide analogs are recently introduced group of peptides with promising results for detection of primary as well as metastatic lesions in malignant melanoma (6, 7). These peptide analogs have the potential of labeling with different radionuclides (8-12) including Technetium-99m and high affinity for melanocortin-1 (MC1) receptors existing on melanocytes specifically and overexpressed in melanoma (6). While in vitro and in vivo animal studies using radiolabeled α-MSH are very promising, there is no human study to address the detectability rate of this new imaging technique. 

This study is aimed to determine the diagnostic value of ^99m^Tc-α-MSH in primary and metastatic malignant melanoma lesions. 

## Methods

***Study population***

Twenty one consecutive patients (13 men) aged 27-80 years, mean±standard deviation (SD) 59.1±14.9 years with pathologically confirmed malignant melanoma with or without metastatic lesions were included in this prospective study. The study was approved by the ethics committee of Tehran University of Medical Sciences and written informed consent was obtained from all patients following a detailed oral and written explanation. Demographic data was precisely obtained and all information related to cancer status including tumor type, primary site, imaging assessment data (including CT and MRI reports), any treatment received after diagnosis and the interval between tumor resection or biopsy and patient’s referral have been collected. Pregnancy, breast feeding and time interval less than 3 weeks from surgical manipulation (including biopsy) and patient’s referral were considered as exclusion criteria.

***Radiopharmaceutical***

The freeze-dried kits of alpha-MSH have been provided by Atomic Energy Organization of Iran (AEOI), Iran. Tc-99m was obtained from a commercially available ^99^Mo/^99m^Tc generator (PARSTEC II, Pars Isotope Company, Iran) and ^99m^Tc-α-MSH was prepared according to the previously reported method with some modifications (7). Briefly, radiolabeling was performed by adding 0.5 mL 0.9 % saline to the kit formulation, allowing the mixture to pre-incubate for 5 min. Then, 25–35 mCi (925-1295 MBq) of ^99m^TcO_4_^-^ in 1 mL normal saline was added. The solution in the vial was incubated at 95°C in a digital dry heater (Hot Pot, Eczacıbaşı-Monrol, Turkey) for 10 min and subsequently cooled down to room temperature for 20 min. The radiochemical purity of the radiolabeled peptide was assessed by instant thin layer chromatography (ITLC) under aseptic condition. Typical labelling efficiencies should be greater than 95%. 

***Patient preparation, image acquisition and reconstruction***

20-30 mCi (740-1110 MBq) of freshly prepared ^99m^Tc-α-MSH was administered intravenously and whole body scans were performed in anterior and posterior projections, 20, 120 and 240 minutes post injection with a dual-head gamma camera (E-cam, Siemens, Malvern, Pennsylvania, USA) equipped with a low-energy high-resolution parallel-hole collimator using a 256×1024 matrix centered on 140 keV with 20% window. The patients were evaluated for any possible side effects including flushing, dyspnea, abdominal discomfort, fever, etc., for the next 48 hours. The scans were interpreted by two nuclear medicine specialists independently who were blind to patients’ conventional imaging data. Both qualitative and semi-quantitative assessments were done. For qualitative assessment, the intensity of abnormal tracer activity was compared with the liver uptake and categorized into three grades of mild (less than liver), moderated (almost equal to liver) and severe (significantly more than liver) uptake, respectively. For semi-quantitative analysis, a region of interest (ROI) was drawn on the abnormal focus of tracer accumulation and the same ROI was drawn on the mediastinum as the background region. The target to background ratio (T/BG) was calculated using the mean count activity of the mentioned regions. The gold standard was pathology in addition to patient’s conventional imaging results. If the scan showed a focal lesion which was not reported as a zone of tumoral involvement in previous assessments, further evaluation was done.

***Statistical analysis***

Using SPSS software (version 16.0; SPSS Inc.) for data analysis, the sensitivity, specificity, negative predictive values (NPV), positive predictive values (PPV) and accuracy were calculated. Quantitative variables were expressed as mean±SD.

## Results

No minor or major complication was seen after ^99m^Tc-α-MSH administration. Foot was the most common location of primary tumor constituted of 12 out of 21 primary locations following with scalp, hand, eyelid, nasal mucosa, arm and trunk. The mean diameter of the primary lesion was 3.3 cm (Mean±SD: 3.3±4.5) ranging from 0.5 to 15. The depth of primary tumors obtaining from pathology reports was between 1 and 30 mm, with the average of 6 mm. In 8 patients the subclass of malignant melanoma was mentioned in pathology report including 4 with acral lentiginous, 3 with nodular and 1 with sinonasal variant. Table 1 shows the distribution of abnormal zones of tracer accumulation in the whole body scans. 

**Figure 1:**
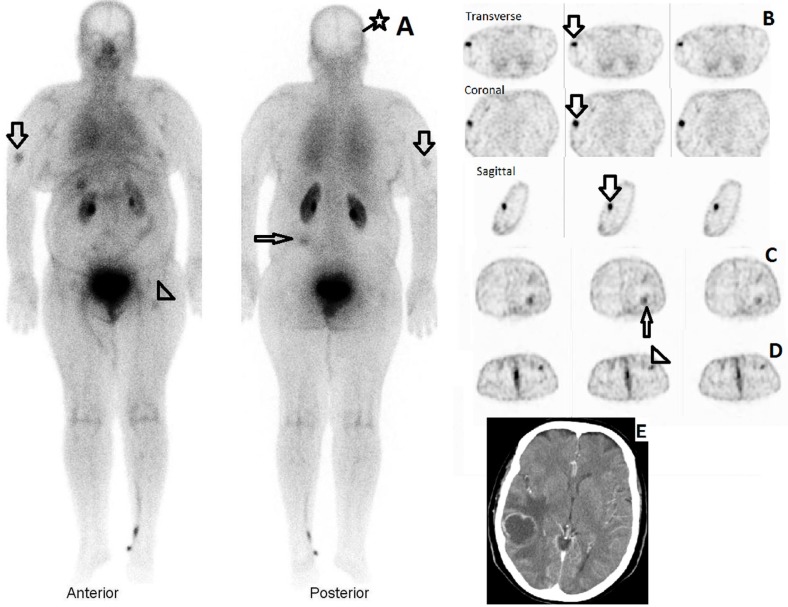
^99m^Tc-α-MSH scan in a 57 years old woman with history of complete resection of right hand malignant melanoma. (A) Two hour whole body scan showed ^99m^Tc-α-MSH localization in the right arm soft tissue (large arrow), left posterior flank soft tissue (small arrow) and right parietal lobe (star arrow). Please note partially infiltrated radiotracer injection site in the left foot and ankle. The activity in the left inguinal region (arrow head) was secondary to infiltrated radiotracer injection and proved to be non-malignant on follow up ultrasonographic evaluation. Physiologic activity in blood pool, kidneys, urinary bladder and gall bladder are also noted. (B) Corresponding SPECT images of the right arm in transit lesion. (C) Transverse SPECT images of the abdominal region. The arrow indicates foci of tracer accumulation in the left posterior flank soft tissue metastasis (D) Transverse SPECT images of the pelvis. The left inguinal uptake secondary to left foot infiltrated radiotracer injection is indicated. (E) Transverse CT images of the right parietal lobe metastasis

The T/BG ratio for the primary tumor bed was 2.51±2.26, 2.56±2.48 and 1.92±1.79 in the whole body scans 20, 120 and 240 minutes post injection, respectively. Figure 1 shows ^99m^Tc-α-MSH whole body scan two hours post injection in a case of malignant melanoma with tracer uptake in distant metastatic lesions as well as locoregional lymph nodes. 

Four out of 21 patients had complete resection of the primary tumor. Among the remained 17 cases, 13 patients showed tracer activity in the tumor site (76%) which was moderate in 11 and severe in two of patients respectively, according to qualitative assessment criteria. However, in one of them it was considered as a false positive finding as the primary tumor had been completely resected prior to imaging. Consequently, the sensitivity, specificity, NPV and PPV were 75%, 80%, 50% and 92%, respectively. 

Twelve patients had proved lymph node involvement consisted of 8 patients (67%) with locoregional and 4 (33%) with distant lymph node involvement. Based on tracer accumulation in the lymph node region, a sensitivity of 92%, specificity of 89%, NPV of 89% and PPV of 92% were achieved. 

The performed imaging modalities before ^99m^Tc-α-MSH scan showed evidence of distant metastasis in 8 patients. The location was lung [1], liver [4], brain [2], lumbar soft tissue [1], chest wall [1] and adrenal gland [1]. Six patients out of 8 with distant metastasis did not show any tracer accumulation corresponding to the metastatic lesions. In the two remained cases intense focal activity was seen. Accordingly, sensitivity, specificity, NPV and PPV of ^99m^Tc-α-MSH for detection of distant metastasis were 25%, 100%, 68% and 100%, respectively. 

## Discussion

In the present study we examined the diagnostic significance of ^99m^Tc-α-MSH in malignant melanoma. To the extent of our knowledge, this is the first study to assess ^99m^Tc-radiolabeled α-MSH analogue imaging in human. Previous animal imaging studies showed promising results with this melanoma-specific agent. The underlying reason being the high affinity of α-MSH peptide analogues for melanocortine-1 (MC1) receptors on melanocytes which is over expressed in malignant melanoma cells (13).

**Table 1:** The distribution of abnormal zones of ^99m^Tc-α-MSH accumulation in the whole body scans

	**No of patients**	**Percent (%)**
**No abnormal uptake**	1	4.76
**Only primary tumor **	6	28.57
**Primary tumor + Regional lymph node**	7	33.33
**Primary tumor + Regional lymph node + Distant area**	1	4.76
**Only regional lymph node**	5	23.80
**Multiple distant metastasis**	1	4.76

Our study showed high PPV of ^99m^Tc-α-MSH for primary tumors. While the first line diagnosis technique in suspicious lesions for melanoma is tissue biopsy, regarding the high PPV, this imaging modality might be an excellent additional method to assess the completion of tissue resection post-operatively. 

As the clinical studies on ^99m^Tc-α-MSH are very limited, the optimal protocol of acquisition and the best time of imaging are still under much debate. A recent animal biodistribution study showed a significantly higher uptake of this radiotracer in metastatic melanoma lesions of the lung compared to the normal lung tissue in both 2h and 4h post injection periods (14). However, the lesion to normal tissue uptake was higher at 2h. Our study showed the same result with highest target to background uptake at 2h images (2.56±2.48). However, the persistency in radionuclide to target binding within the first two hours is a favorable characteristic of this agent which makes the scheduling time more flexible for nuclear medicine department and as a result more convenient for the patients. 

The current study showed high sensitivity of ^99m^Tc-α-MSH imaging for both primary lesion and lymph node involvement (75% and 92%); however, the detection rate for distant metastasis was low (25%) which is remarkably less than conventional imaging techniques such as MRI with sensitivity of 86.9-100% for brain metastasis (15), computed tomography (CT) with 93% sensitivity for pulmonary metastasis (16) and 94% detection rate of ^18^F-FDG PET/CT for distant metastasis of malignant melanoma (17). However, since this scan showed a high specificity in distant metastasis, it can be used as a complementary method in suspicious lesions throughout the body.

Hybrid imaging has added value for better lesion localization and discrimination between physiologic uptake and pathologic localization of radiotracer and the diagnostic value of ^99m^Tc-α-MSH SPECT/CT in lesion detection in comparison with whole body scan may serve as a clue to determine the optimal imaging protocol for this new modality. Moreover, CT component of SPECT-CT acquisition could reveal tumoral lesions without significant radiotracer uptake.

 Regarding the specific affinity of α-MSH peptide analogs for MC-1, radiolabeling of this agent with beta emitters has been suggested as a promising treatment modality for chemotherapy resistant melanomas (14, 15). As a confirmatory pre-treatment imaging, ^99m^Tc-α-MSH may serve to predict the response rate following radionuclide therapy. These concepts however, requires further/additional larger scale studies to be conducted to validate our findings in this limited study. 

## Conclusion

^99m^Tc-α-MSH is a newly introduced agent for diagnosis of tumoral lesions in malignant melanoma with high sensitivity for primary lesions and local lymph node involvement. Our preliminary results are promising to suggest this targeted imaging modality as a new complementary method in management of malignant melanoma. We recommend further evaluation on diagnostic value of ^99m^Tc-α-MSH with larger patient population and a head to head comparison with ^18^F-FDG PET/CT in future studies. 
